# Preditores Clínicos e Laboratoriais do Desenvolvimento de Valvopatias na Doença Renal Crônica: Uma Revisão Sistemática

**DOI:** 10.36660/abc.20240222

**Published:** 2025-08-14

**Authors:** Hayala Machado Cavalcante Conceição, Ana Luísa Vaz Valois, Erick Magalhães Silva Ramos, Ana Marice Ladeia

**Affiliations:** 1 Escola Bahiana de Medicina e Saúde Pública Salvador BA Brasil Escola Bahiana de Medicina e Saúde Pública, Salvador, BA – Brasil; 2 Universidade Salvador Salvador BA Brasil Universidade Salvador, Salvador, BA – Brasil

**Keywords:** Doenças das Valvas Cardíacas, Insuficiência Renal Crônica, Fatores de risco

## Abstract

**Fundamento:**

A doença renal crônica (DRC) está associada à maior prevalência de valvopatias e à maior mortalidade por causas cardiovasculares. Aspectos que exercem influência sobre a gênese da calcificação valvar cardíaca (CVC) nestes pacientes não estão bem definidos.

**Objetivo:**

Determinar fatores de risco à calcificação valvar em pacientes com DRC.

**Métodos:**

Revisão sistemática baseada no PRISMA, que incluiu estudos observacionais avaliando a associação entre aspectos clínicos e laboratoriais e CVC em pacientes com DRC, realizando ou não hemodiálise ou diálise peritoneal. Os artigos foram obtidos em bases de dados (MEDLINE; SCIELO; CENTRAL; EMBASE; LILACS/BVS) e selecionados por dois autores de forma cega; um terceiro atuou em discrepâncias. Coleta e síntese dos dados foi realizada pela autora principal. Avaliação da qualidade metodológica e risco de viés embasados no STROBE e Newcastle-Ottawa.

**Resultados:**

Foram encontrados 783 estudos, dos quais 20 foram incluídos, englobando 13 314 pacientes em 10 países. Os aspectos mais fortemente associados à CVC foram idade > 55 anos, taxa de filtração glomerular < 53mL/min/1,73m^2^, terapia renal substitutiva (TRS) > 20 meses, hipoalbuminemia, proteína C reativa (PCR), níveis séricos de IL-6, TNF- α, paratormônio, hiperfosfatemia, hipercalcemia, produto Ca x P e FGF-23 decorrentes do hiperparatireoidismo secundário. Foram estudadas valvas mitral e aórtica. Não houve diferença entre hemodiálise versus diálise peritoneal.

**Conclusão:**

Idade, TRS, inflamação crônica e hiperparatireoidismo favorecem a deposição de Ca e P nas válvulas, tornando os pacientes com DRC mais susceptíveis à CVC. O acompanhamento de tais parâmetros permite oportunidades de prevenção e tratamento.

**Figure:**
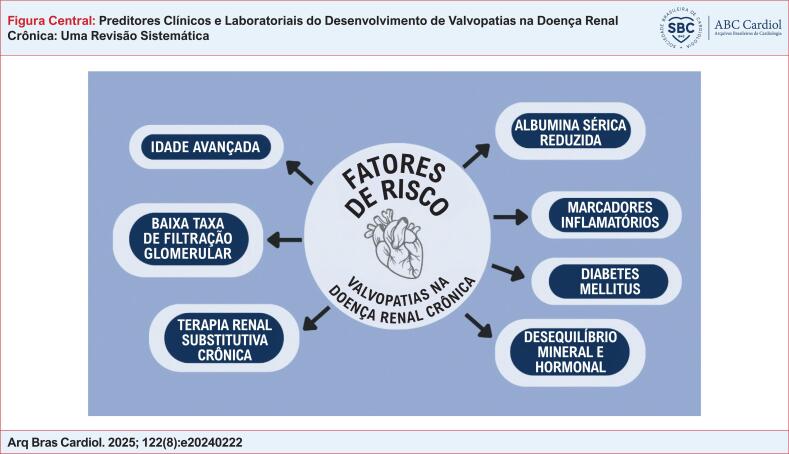


## Introdução

Causas cardiovasculares correspondem a mais de 50% dos óbitos em pacientes com doença renal crônica (DRC) em comparação à população geral,^1^ e a prevalência de valvopatias é significativamente maior neste grupo.^2^ Anormalidades valvulares, principalmente em válvulas aórtica e mitral, bem como necessidade de hemodiálise (HD) ou diálise peritoneal (DP) são marcadores de morbimortalidade. Entretanto, esse é um dos aspectos menos abordados no estudo da DRC, tendo sua relevância ignorada por muitos profissionais.

A patogênese das valvopatias envolve processos inflamatórios (sistêmicos e locais), ação modificada do hormônio paratireoidiano (PTH) no hipertireoidismo secundário,^3 – 5^ desordens no metabolismo cálcio-fósforo,^5 , 6^ tipo (HD ou DP), duração da diálise,^3 , 7 , 8^ níveis séricos de colesterol,^3 , 9^ albumina,^3 , 9^ além da volemia.^9^ Entretanto, os aspectos que de fato estão envolvidos na patogênese da calcificação valvar e valvopatias em pacientes com DRC, e que justifiquem a maior prevalência de tais condições neste grupo, não estão bem definidos.^2^

O objetivo desta revisão sistemática é identificar possíveis preditores clínicos e laboratoriais que constituam fatores de risco para a calcificação valvar em indivíduos com DRC que estejam ou não realizando HD ou DP.

## Métodos

A revisão sistemática foi conduzida de acordo com o *Preferred Reporting Items for Systematic Reviews and Meta-Analysis* para protocolos (PRISMA-P) e submetido ao registro no *Prospective Register of Systematic Reviews (PROSPERO)* (CRD42021291576).

### Estratégia de busca

A estratégia de busca utilizou DeCS ( *Descritores em Ciências da Saúde* ) e MeSH Terms ( *Medical Subject Readings* ), nas plataformas MEDLINE ( *Medical Literature Analysis and Retrieval System* ), LILACS ( *Literatura LatinoAmericana e do Caribe em Ciências da Saúde* ) *,* SCIELO ( *Scientific Electronic Library Online* ), Biblioteca Cochrane (CENTRAL) e EMBASE. A busca com MeSH Terms foi a seguinte: ((((((End-Stage Kidney Disease) OR (Chronic Kidney Failure) OR (End-Stage Renal Disease) OR (Chronic Renal Failure) OR (End-Stage Renal Failure)))))) OR (Dialysis) AND ((Risk Factor) OR (Risk Factors)) AND ((Heart Valve Disease) OR (Valvulopathy)) AND (Calcification). A busca com termos DeCS foi (Insuficiência Renal Crônica) OR (Doença Renal Crônica) AND (Valvulopatia) OR (Valvopatia).

### Critérios de elegibilidade

Estudos observacionais, em português, inglês e espanhol, que incluíssem pacientes com DRC (com ou sem diálise) e avaliassem a associação entre aspectos clínicos/laboratoriais e calcificação valvar foram considerados elegíveis. Estudos com dados incompletos ou que não atenderam a todos os critérios foram excluídos.

### Identificação e seleção de estudos

Dois autores realizaram a seleção de artigos no programa *Rayyan QCRI* , e um terceiro atuou nos casos de discordância. O *Teste de Concordância Kappa (K)* avaliou a concordância dos revisores. Referências de artigos relevantes foram verificadas e o fluxograma PRISMA resumiu as etapas de seleção.

### Extração e análise de dados

A coleta de dados foi realizada pela autora principal em formulário eletrônico baseado no *Critical Appraisal and Data Extraction for Systematic Reviews of Prognostic Factor Studies (CHARMS-PF Checklist)* .

A qualidade metodológica e o risco de vieses foram avaliados pelo *The Strengthening the Reporting of Observational Studies in Epidemiology* ( *STROBE)* e a escala *Newcastle-Ottawa (NOS)* , respectivamente. Os estudos incluídos apresentaram desempenho moderado nas análises, conforme demonstrado nas tabelas a seguir.

## Resultados

### Seleção dos estudos

**Figura 1:**
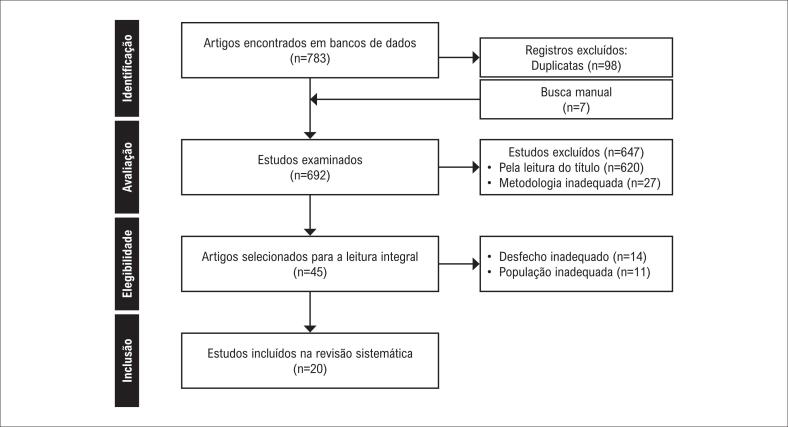
Fluxograma dos estudos avaliados nesta revisão.

Foram identificados 790 artigos, sendo 783 por bancos de dados e sete por busca manual e listas de referências. Após remoção de duplicatas e aplicação dos critérios de elegibilidade, 20 artigos foram selecionados ( Figura 1 ), sendo 13 de metodologia transversal, seis coortes e um caso-controle, englobando 13 314 pacientes em 10 países, com amostras variando de 30 a 3929 participantes. A concordância entre os examinadores, na seleção dos artigos, apresentou um valor de Kappa > 0,758 (boa concordância).

Alguns estudos avaliaram pacientes com DRC sem categorização dos estágios da doença (que variam de 01 a 05 pela literatura),^4^ e outros estudos incluíram apenas pacientes em determinados estágios. Houve estudos que incluíram pacientes sob realização de terapia renal substitutiva (TRS) e outros que avaliaram especificamente HD ou DP. Os artigos incluídos empregaram análises multivariadas e adotaram metodologias distintas.

A Tabela 1 resume as principais características dos 20 estudos incluídos, com informações sobre amostras populacionais e pontuação obtida por cada um deles.

**Tabela 1:** Características gerais dos estudos incluídos na revisão sistemática, amostras populacionais e pontuação na análise de qualidade

Autores, ano e país	Desenho do estudo	Descrição da amostra (n)	Desfecho avaliado	Sexo Masculino (%)		Média de idade (anos)		STROBE	Newcastle-Ottawa
Alamir et al, 2015, EUA^10^	Transversal	DRC leve a moderada (n=2070)	CAM; TC não contrastada	53,7%		58 (21-74)		86,36%	4
Asselbergs et al, 2008, EUA^20^	Coorte retrospectiva	DRC (n=3929)	CAM, CAA e EVA; ECO-2D	60%		74 ± 5		90,9%	5
Avila-Díaz et al, 2013, México^14^	Coorte prospectiva	DRC sob DP (CAPD ou DPA) (n=124)	CVC geral; ECO-2D	68,5%		46 (30-54)		95,45%	7
Chen et al, 2021, China^25^	Caso-controle	DRC estágios 2 a 5 (n=180)	CVC geral; ECO-2D	63%		58 ± 14		86,36%	2
Di Lullo et al, 2015, Itália^26^	Transversal	DRC estágios 3 e 4 (n=100)	CVC geral; ECO-2D	60%		51 (46-56)		72,72%	4
Engole et al, 2020, República Democrática do Congo^15^	Transversal	DRC sob HD (n=60)	CVC geral; ECO-2D	71,6%		52,5 ± 15,9		72,72%	6
Fernández et al, 2021, Espanha^11^	Coorte prospectiva	DRC estágios 2- 5, sob TRS ou não (n=397)	CVC geral; ECO-2D	61%		59,1 ± 11,5		72,72%	5
Fox et al, 2006, EUA^22^	Transversal	DRC (n=3047)	CVC geral; ECO-2D	47,6%		59 ± 10		90,9%	5
Genctoy et al, 2015, Turquia^38^	Transversal	DRC sob HD (n=76)	CVC geral; ECO-2D	64,47%		60,5 ± 15,5		77,27%	6
Guerraty et al, 2015, EUA^12^	Transversal	TFG 20-70 ml/min/1,73m^2^ e CVA (n=1923)	CVA; TC não contrastada	53%		58,45 ± 11,45		90,9%	7
Guo et al, 2020, China^16^	Transversal	DRC sob HD (n=145)	CVC geral; ECO-2D com Doppler	54,5%		50 (23-74)		81,8	5
Hoshina et al, 2011, Japão^27^	Coorte retrospectiva	DRC sob HD (n=30)	Estenose aórtica; ECO-2D	EAPR (n=30): 40%	EAPL (n=30): 53%	EAPR: 73,6 ± 6,1	EAPL: 69,8 ± 7,5	81,81%	6
Ikee et al, 2010, Japão^17^	Transversal	DRC sob HD (n=112)	CVC geral; ECO-2D	68,7%		67 ± 10		86,36%	6
Plytzanopoulou et al, 2020, Grécia^18^	Transversal	DRC sob HD (n=42)	CVC geral; ECO-2D	69%		72,97 ± 11,6		95,45%	5
Rong et al, 2018, China^13^	Transversal	DRC (n=288)	CVC geral; ECO-2D	65,9%		CVC: 70,42±11,83;	Sem CVC: 56,47±15,00	81,81%	3
Sayarlioglu et al, 2013, Turquia^19^	Transversal	DRC sob HD (n-129)	CVC geral; ECO-2D	CVC: 52,3%	Sem CVC: 46,5%	CVC: 48,2 ± 16,8	Sem CVC: 60,3 ± 13,5	77,27%	6
Silva et al, 2022, Portugal^23^	Transversal	Diabéticos com DRC estágios 2- 4 (n=80)	CVC geral; ECO-2D	71,3%		56 ± 8,1		81,81%	6
Tian et al, 2016, China^3^	Coorte prospectiva	DRC sob DP (n=194)	CVC geral; ECO-2D	54%		61,6 ± 12,5		90,9%	7
Usuku et al, 2019, Japão^24^	Coorte retrospectiva	DRC sob HD (n=95)	CAM; ECO-2D	CAM (n=28): 54%	Sem CAM (n=67): 54%	CAM (n=28): 65 ± 10,7	Sem CAM (n=67): 62,6 ± 13,2	86,36%	7
Xiong et al, 2022, China^21^	Transversal	DRC estágios terminais, sob HD (n=293)	CVC geral; ECO-2D, TC e/ou AngioTC em alguns casos	54,6%		64 ± 7,0		77,27%	4

AngioTC: angiotomografia computadorizada; CAM: calcificação do anel mitral; CAA: calcificação do anel aórtico; CVC: calcificação de válvula cardíaca; capd: diálise peritoneal contínua ambulatorial; DPA: diálise peritoneal automática; DP: diálise peritoneal; DRC: doença renal crônica; EAPR: Estenose Aórtica de progressão rápida; EAPL: estenose aórtica de progressão lenta; EVA: esclerose do anel aórtico; ECO-2D: ecocardiograma bidimensional; HD: Hemodiálise; TC: tomografia computadorizada; TRS: terapia renal substitutiva; o nível de significância estatística foi p < 0,05, exceto para o estudo de Engole et al.,^15^ que não determinou o valor em seus métodos.

### Síntese dos resultados

Foram identificados 38 fatores de risco para calcificação valvar. A Tabela 2 resume os estudos que avaliaram fatores de risco em pacientes com DRC em geral. A Tabela 3 mostra os resultados dos estudos que incluíram apenas pacientes sob TRS, seja HD ou DP. Os resultados estão condensados na Figura Central.

**Tabela 2:** Fatores de risco identificados em estudos que incluíram pacientes com doença renal crônica em geral

Autores, ano e país	Desfecho avaliado e método de definição	Fatores de risco identificados no estudo	Calcificação valvar e/ou valvopatias encontradas
Alamir et al, 2015, EUA^10^	CAM em duas TCs pelo algoritmo de Agatston.	CAM associada a TFG < 50ml/min/1,73m^2^ (ORa = 2,30, IC95% 1,40-3,79), idade > 55 anos (OR = 4,01, IC95% 2,55-6,32), fosfato > 4,1 mg/dl (OR = 3,33, IC95% 1,37-8,09)	Prevalência CVM: 331 (16%).
Asselbergs et al., 2008, EUA^20^	CAM e CAA; hiperecogenicidade em ECO-2D; espessamento da cúspide aórtica e velocidade de fluxo <2,0m/s.	TFG <45 mL/min/1,73 m^2^ (OR =1,15, IC95% 1,03-1,12, p=0,015) e cistatina C (OR = 1,12; IC95% 1,03-1,23, p=0,013) associados a prevalência de CAM	CAM leve: 1495, moderada: 133 grave: 12. CAA: leve: 1698, moderada: 49, grave: 1. Esclerose aórtica em 2114.
Chen et al., 2021, China^25^	CVC geral, massas hiperecogênicas de diâmetro ≥1 mm em ECO-2D Doppler.	CVC associada creatinina sérica (DRC5, 845,09 ± 334,18 umol/L, p<0,05), FGF-23 (DRC5, 1039,43 ± 214,83 pg/ml, p<0,05) e proteína Klotho (DRC2-3, 159,05 ± 27,53 U/L, p<0,05) Pacientes em estágio 5 possuíram maiores níveis de Hb (90,52 ± 23,36 g/L, p<0,05), albumina (36,61 ± 4,37 g/L, p<0,05), osteocalcina (197,32 ± 78,88 ng/ml, p<0,05), PTH (392,40 ± 233,88 pg/ml, p=0,05) e FA ósseo-específica (86,36 ± 18,18 U/L, p=0,05) e menores níveis de cálcio sérico corrigido (2,06 ± 0,15 mmol/L, p<0,05).	CVC total: 41 (22,7%); 4 pacientes em DRC 2-3; 12 pacientes em DRC4, 25 pacientes em DRC5.
Di Lullo et al., 2015, Itália^26^	CVC geral por Escore de Wilkins em ECO-2D.	CVA e PTH (r2 = 0,212 p=0,03) e FGF-23 (r2 = 0,272; p=0,01). Escore de CVM associado ao cálcio sérico (r2 = 0,565; p=0,01)	CVC total: 100%. CVM: 96; 61 em escore 1, 34 em escore 2, 1 em escore 3. CVA: 100 (100%).
Fernández et al., 2021, Espanha^11^	CVM massas > 5mm e CVA > 2mm, em ECO-2D.	CVM associada a idade (OR = 1,05. IC95% 1,03-1,08), doença vascular periférica (OR = 4,22, IC95% 1,53-13,12) e produto Ca x P (OR = 1,03, IC95% 1,01-1,05) CVA associada a idade (OR = 1,10, IC95%1,05-1,15), níveis de P (OR = 1,64, IC95% 1,16-2,42), aterosclerose e maior área total de placa carotídea (OR = 3,10, IC95% 1,18-8,80)	CVM aumentou de 96 (24,2%) para 123 (31%). CVA Aumentou de 119 (30%) para 171 (43,1%).
Fox et al., 2006, EUA^22^	CVC geral; CVM se massas hiperecogênicas > 0,3cm no Modo M ou mais de 1/3 do anel na janela paraesternal. CVA se mais de ½ do anel hiperecogênico.	CVC associada a HAS (67%, p<0,01), DM (21%, p<0,05), colesterol total/HDL (4,8 ± 1,8, p<0,01) e IMC (29,2 ± 5,2 kg/^2^, p<0,001); TFG significativamente menor nos pacientes com CVC (TFG = 78 ± 25ml/min/1,73m^2^, p<0,001)	CVC total: 284 (9,3%). CVM: 130 (4,3%). CVA: 112 (3,7%). EVA: 188 (6,2%).
Guerraty et al, 2015, EUA^12^	CVA pela pontuação do algoritmo de Agatston, TC não contrastada.	Associação independente entre CVA moderada/grave e idade (respectivamente, 62,26 ± 7,9 e 66,53 ± 7 p<0,001), IMC (31,73 e 31,72, p<0,0007), CA (105,01 ± 15,53 e 106,81 ± 14,31, p<0,001), PAS (127,75 ± 21,99 e 132,28±21,21, p <0,001), hipercolesterolemia (463/90% e 401 94%, p<0,001), HbA1c (6,73 ± 1,52% e 6,64 ± 1,36%, p<0,001), e MET reduzido (198,37 ± 25,59 e 178,24 ± 120,95, p<0,001) e DM (281/55% e 52/59%, p<0,001), HAS (478/93% e 409/96%), p<0,001 ou doença cardiovascular (149/29% e 176/41%, p<0,001); PCR (4,85 ± 7,29 e 5,48 ± 8,77, p=0,0100), homocisteína plasmática total (14,17 ± 4,86 e 16,55 ± 7,06, p<0,001) e ALP(a) (100,84 ± 34,99 e 99,7± 34,23), p=0,0041. TFG média foi de 44,6 ml/min/1,73 m^2^ e foi associada a CVA (p<0,0001).	CVA leve a moderado: 515 (26,8%) CVA grave: 426 (22,1%).
Rong et al., 2018, China^13^	CVC total, massas hiperecogênicas > 1mm em ECO-2D com Doppler.	CVC associada a idade (70,42 ± 11,83 anos, p<001; OR = 1,091, IC95% 1,048-1,136, p<0,05), níveis menores de pré-albumina (238,44 ± 91,48 g/L, p=0,05), colesterol (OR = 0,488, IC95% 0,306-0,780, p=0,03), TG (1,4 ± 0,65 mmol/L, p=0,37) e APO-E (37,3, de 30,2-45,6 mg/L, p=0,09), LDL (OR = 163,028, IC95% 3.796-7002.467, p=0,08), PCR (5,5, de 0,5-16,35 mg/L, p=0,04) e IL-6 (18,76, de 5,95-46,9 pg/mL, p=0,05). Grupo com CVC em estágios mais avançados da DRC (em estágio 5 sob diálise, 30,3% com CVC vs. 18,9% sem CVC, p=0,048).	CVC total: 66 (22,9%). CVM: 14 (21,2% dos 66); CVA: 100% dos 66).
Silva et al., 2022, Portugal^23^	CVC total; CVM pelo Escore de Wilkins e CVA com Escore proposto por Lullo et al, em ECO-2D.	CVM e CVA possuíam menores TFGe (p< 0,0001), GRP (p< 0,0001), Mg (p= 0,029 para CVM e p= 0,001 para CVA) e α Klotho (p= 0,002), e níveis mais elevados de P (p= 0,001), PTH (p= 0,025 para CVM e p=0,030 para CVA), FGF-23 (p< 0,0001) e TNF-α (p=0,037). Houve correlação negativa entre GRP e CVM (r =-0,754, p< 0,0001) e níveis baixos são fatores de risco independente para CVM (ORa = 0,268, 95% IC 0,101-0,725, p= 0,005; aPR = 0,750; IC 95% 0,456–0,976; p= 0,024) e CVA (ORa = 0,202, IC95% 0,109-0,401, p= 0,022; aPR = 0,813; IC 95% 0,113–0,937; p< 0,0001). O mesmo para baixos níveis de Mg e CVM (ORa = 0,747, IC95% 0,263-0,921, p= 0,003; aPR = 0,762; IC95% 0,256–0,963; p = 0,02) e CVA (ORa = 0,580, IC95% 0,173-0,948, p= 0,008; aPR = 0,809; IC95% 0,391–0,974; p= 0,006). Também são fatores de risco altos níveis de P (para CVM, ORa = 1,078, IC95% 1,0-1,612, p= 0,001; para CVA, ORa = 1,497, IC95% 1,004-2,378, p= 0,002) e FGF-23 (para CVM, ORa = 1,209, IC95% 1,099-1,619, p= 0,035; para CVA, ORa = 1,126, IC95% 1,034-1,436, p= 0,011).	CVM: 29 (36,2%); CVA: 29 (36,2%).

FA: fosfatase alcalina; APO-E: apolipoproteína E; aPR: adjusted prevalence ratio CAPD: diálise peritoneal ambulatorial contínua; CA: Circunferência abdominal; CAM: calcificação do anel mitral; CAA: calcificação do anel aórtico; CVC: calcificação da valva cardíaca; CVA: calcificação da valva aórtica; CVM: calcificação da valva mitral; DM: diabetes mellitus; DRC: doença renal crônica; DPA: diálise peritoneal automatizada; ECO-2D: ecocardiograma Bidimensional; EVA: esclerose do anel aórtico; FGF-23: fator de crescimento de fibroblastos 23; GRP: proteína Gla-Rich; HAS: hipertensão arterial sistêmica; Hb: hemoglobina; HbA1c : hemoglobina glicada; HDL: lipoproteína de alta densidade; IMC: índice de massa corporal; ITB: índice tornozelo-braquial; LDL: lipoproteína de baixa densidade; MET: "metabolic equivalent of task". Mg: magnésio; OR: odds ratio; ORa: odds ratio ajustado; P: fósforo; PAS: pressão arterial sistólica; PCR: proteína C Reativa; PTH: paratormônio; TC: tomografia computadorizada; TFG: taxa de filtração glomerular; TG: triglicerídeos; TNF-α: fator de necrose tumoral alfa; TRS: terapia renal substitutiva; o nível de significância estatística foi p < 0,05.

**Tabela 3:** Fatores de risco identificados em estudos que incluíram apenas pacientes com DRC sob terapia renal substitutiva

Autores, ano e país	Desfecho avaliado e método de definição	Fatores de risco identificados no estudo	Calcificação valvar e/ou valvopatias encontradas
Avila-Díaz et al., 2013, México^14^	CVC geral, ecos brilhantes > 1 mm; ECO-2D.	CVM associada a idade (RR=1,051, IC95% 1,06-1,09, p=0,02), DM (RR=0,287, IC95% 0,1-0,8, p=0,01), osteoprotegerina (RR=1,15, IC95% 1,03-1,21, p=0,008), PTH (RR=0,41, IC95% 1,08-50,5, p=0,04) e PCR (RR=1,09, IC95% 1,04-1,19, p=0,04). iPTH (RR = 2.002, IC95% 1,052–3,81, p<0,034) para CVA.	CVC total: 57 (46%); CVM: 15 (26,3%); CVA: 33 (57,8%); calcificação em ambas as válvulas: 9 (15,8%).
Engole et al., 2020, República Democrática do Congo^15^	CVC geral, ecos brilhantes > 1 mm; ECO-2D com Doppler.	Idade > 60 anos (ORa = 4,48; IC95% 1,67-30,10, p=0,003), tabagismo (ORa=4,57; IC95% 1,15-13,36, p=0,016), níveis de P (ORa=2,17; IC95% 1,83-5,65, p=0,012) e hipertensão (ORa=3,963, 95% IC 1,24–15,7, p=0,014) foram independentemente associados a CVC.	CVC total: 23 (38%); desses, CVM: 23%; CVA: 64%.
Genctoy et al, 2015, Turquia^38^	CVC geral, ecos brilhantes > 1 mm; ECO-2D.	CVC associada a T-PAFT (OR=1.006, não apresenta IC, p=0,04).	CVC total: 50 (65,8%); 18 (23,7%) em uma cúspide e 32 (42,1%) em duas.
Guo et al., 2020, China^16^	CVC geral com calcificação avaliado pelo GCCS; ECO-2D Doppler.	Idade (r = 0,25; p=0,003), duração da hemodiálise (r = 0,27; p=0,001), e iPTH sérico (r = 0,18; p=0,03) e ALP sérica (r = 0,24; p=0,003; OR =3,87, IC95% 1,86–8,07, p<0,001; ORa = 3.92, IC95% 1,37–11,2, p=0,011; em análise por Splines Cúbicos, a probabilidade de GCCS = 1 aumentou significativamente quando FA ultrapassou o valor 232 U/L). Houve correlação negativa com albumina sérica (r = –0,22; p=0,008). FA>232 U/L e idade > 60 anos são determinantes para alto risco de CVC.	CVC GCCS ≥ 1: 83.
Hoshina et al., 2011, Japão^27^	Estenose aórtica definida a partir da progressão do GPP entre dois ECO-2D com intervalo de 3 meses.	Pressão arterial sistólica (161 ± 21,5 mmHg, OR = 1,06, IC95% 1-1,11, p=0,04) e níveis séricos de cálcio (9,66 ± 1,05 mg/dl, OR = 6,08, IC95% 1,28-28,8, p=0,02).	EAPR: 15; EAPL: 15.
Ikee et al, 2010, Japão^17^	CVC geral, ecos brilhantes > 1 mm; ECO-2D.	Idade associada a CVC geral (para CVA, OR = 1,06, IC95% 1,01-1,11, p=0,01. Para CVM, OR = 1,04, IC95% 1,01-1,09, p=0,04). Cálcio associado à CVA (OR = 2,16, IC95% 1,05-4,44, p=0,03) B2-microglobulina sérica associada à CVM (OR = 1,10, IC95% 1,01-1,2, p=0,01) e correlacionou-se com a duração da HD (r = 0,273, p=0,004), albumina sérica (r = 0,209, p=0,02), colesterol total (r = 0,243, p=0,01), triglicerídeo (r = 0,189, p=0,04), HDL-C (r = 0,337, p=0,001), e PCR (r = 0,246, p=0,009).	CVM: 58 (51,7%); CVA: 85 (75%); calcificação em ambas as válvulas: 48 (42,8%).
Plytzanopoulou et al., 2020, Grécia^18^	CVC geral, ECO-2D.	Idade (AUC 0,734, IC95% 0,58–0,89, p=0,011), PCR (AUC 0,692, IC95% 0,516–0,868, p =0,03) diminuição na albumina sérica (AUC 0,73, IC95% 0,57–0,89, p=0,012) são preditores positivos de CVC. Idade >76,5 anos (OR 9,56, IC95% 1,54–59,42, p= 0,015) e PCR>3,5 mg/dl (OR 9,26, IC95% 1,51–56,83), p=0,016) associados a CVC de alto grau.	CVC ausente ou leve em 24 (57,15%); CVC moderada ou grave em 18 (42,85%). CVM: 21 (50%); CVA: 16 (38,10%).
Sayarlioglu et al, 2013, Turquia^19^	CVC geral, ECO-2D.	Idade avançada (60,3 ± 13,5 anos, p<0,001) e baixa albumina sérica (3,7 ± 0,5 mg/dl, p=0,02) foram associadas a calcificação e ambas as válvulas. DM associada a CVM (16,3%, p=0,02). Duração da diálise dos pacientes com CVM e CVA foi significativamente maior do que a de outros grupos (19,6 ± 40,6 meses vs. 7,1 ± 5,8 meses, p=0,01).	CVC total: 43 (33,3%); CVM: 30 (23,3%); CVA: 28 (21,7%).
Tian et al, 2016, China^3^	CVC geral, se ecos brilhantes de > 1 mm; ECO-2D.	CVC associada a DP crônica (OR=1.039, IC95% 1.004-1.075, p=0,03), níveis séricos de P (OR=2.569, IC95% 1.227-5.377, p=0,01), diminuição dos níveis séricos de albumina (OR albumina =0,935, IC95% 0,877-0,997, p=0,01).	CVC total: 97 (32%). CVM: 7 novos casos; CVA: 30 novos casos;
Usuku et al, 2019, Japão^24^	CAM, definida como massa ecodensa ≥ 5mm na junção do sulco atrioventricular e folheto posterior da valva mitral; ECO-2D.	A duração da terapia de hemodiálise foi significativamente maior em pacientes com CAM do que naqueles sem (13,4 ± 8,6 anos vs. 7,7 ± 8,4 anos; p<0,01) e houve associação entre duração da HD e CAM (OR-1,09, IC95% 1,02–1,16; p<0,01). O número de fatores de risco coronariano (HAS, dislipidemia, tabagismo e DM) foi significativamente maior no grupo progressão da CAM do que no grupo não progressão (1,91 ± 0,83 vs. 1,18 ± 0,99; p=0,03) e houve associação entre ambos (OR = 2,67; IC95% 1,24–5,76; p=0,01).	CAM: 28 (29%), com diâmetro transverso do anel mitral = 12,4 ± 7,4 mm.
Xiong et al, 2022, China^21^	CVC geral, por ECO-2D; 282 também realizaram TC. AngioTC se placa calcificada com mais de 130 na TC,	HD há ≥ 36 meses (OR = 2,25; IC95% 1,26-4,02, p=0,006), DM (OR = 1,81, IC95% 1,04-4,12, p=0,037), níveis baixos de albumina sérica (considerando albumina ≥ 40 g/L vs. < 40 g/L com OR = 0,54, IC95% 0,29-0,99 p=0,047), cálcio sérico ≥ 2,11 mmol/L (OR = 2,04, IC95% 1,01-4,12, p = 0,046) e pressão de pulso > 72 mmHg (OR = 3,22, IC95% 1,85-5,59, p<0,001) foram independentemente associados a CVC	CVC total: 93; CVM: 37; CVA: 68; calcificação em ambas as válvulas: 12.

AngioTC: angiotomografia computadorizada; CAM: calcificação do anel mitral; CAPD: diálise peritoneal ambulatorial contínua; CVC: calcificação de válvula cardíaca; DM: diabetes mellitus; DPA: diálise peritoneal automatizada; EAPR: estenose aórtica de progressão rápida; EAPL: estenose aórtica de progressão lenta; FA: fosfatase alcalina; GCCS: sistema global de pontuação de cálcio cardíaco; GPP: gradiente de pico de pressão; HAS: hipertensão arterial sistêmica; HD: hemodiálise; PP: pressão de pulso; PCR: proteína C Reativa; T-PAFT: tecido adiposo periaórtico torácico; aOR: adjusted odd ratio; o nível de significância estatística foi p < 0,05, exceto para o estudo de Engole et al.,^15^ que não determina o valor em seus métodos.

O fator associado mais frequente foi idade avançada, presente em 10 publicações.^10 – 19^ Para Alamir et al.,^10^ houve associação positiva a partir dos 55 anos (OR = 4,01, IC95% 2,55-6,32). Observou-se associação acima de 60 anos nos estudos de Engole et al.^15^ (ORa = 4,48; IC95% 1,67-30,10; p=0,003) Guo et al.^16^ (r = 0,25; p=0,003), Guerraty et al.^12^ (CVA moderada/grave, respectivamente, 62,26 ± 7,9 e 66,53 ± 7 p<0,001), e Sayarlioglu et al.^19^ (60,3 ± 13,5 anos, p<0,001); e Rong et al.^13^ acima dos 70 anos (70,42 ± 11,83 anos, p<001; OR = 1,091, IC95% 1,048-1,136; p<0,05). Alguns estudos não estabeleceram um valor de referência.^11 , 14 , 16 – 18^

Baixa taxa de filtração glomerular (TFG) na ausência de TRS^10 , 12 , 20 – 23^ foi outro fator associado, sendo TFG < 50mL/min/1,73m^2^ no estudo de Alamir et al.^10^ (ORa = 2,30; IC95% 1,40-3,79), ou menor que 45mL/min/1,73m^2^ no estudo de Asselbergs et al.^20^ (OR =1,15; IC95% 1,03-1,12, p=0,015) e Guerraty et al.^12^ (p<0,0001). Fox et al.^22^ relataram associação com TFG = 78 ± 25mL/min/1,73m^2^ (p<0,001), nível considerado reduzido com base nos valores de TFG normais (90 mL/min/1,73 m^2^),^4^ mas acima do valor de corte determinante de DRC. A cistatina C obteve associação com Calcificação do Anel Mitral (CAM) no estudo de Asselbergs et al.^20^ (OR = 1.12; IC95% 1,03-1,23; p=0,013).

Em pacientes sob HD ou DP, a cronicidade desses procedimentos também foi associada à calcificação valvar cardíaca (CVC).^3 , 16 , 19 , 21 , 24^ Sayarlioglu et al.^19^ observaram que pacientes com CVC estavam realizando HD há mais tempo (19,6 ± 40,6 meses vs. 7,1 ± 5,8 meses em não TRS, p=0,01). Xiong et al.^21^ obtiveram associação significativa entre CVC e HD há mais de 36 meses (OR = 2,25; 95%IC 1.26-4.02, p=0,006,). Usuku et al.^24^ demonstraram resultado semelhante para calcificação da valva mitral (CVM), comparando grupos que iniciaram a TRS há mais tempo (13,4 ± 8,6 anos vs. 7,7 ± 8,4 anos; OR-1,09; IC95% 1,02–1,16; p<0,01). Tian et al.^3^ analisaram especificamente DP, obtendo associação positiva para duração superior a 20 meses (OR=1,039; IC95% 1,004-1,075, p=0,03).

Níveis reduzidos de albumina sérica foram associados a CVC em diversos estudos,^3 , 13 , 16 , 18 , 19 , 21 , 25^ com destaque para pacientes sob TRS. Rong et al.^13^ associaram a CVC a níveis de pré-albumina entre 238.44 ± 91.48 g/L, p=0,05. Para Plytzanopoulou et al.,^18^ essa diminuição é um preditor positivo de CVC, considerando a AUC 0,73, IC95% 0,57–0,89, p=0,012. Chen et al.^25^ identificaram maiores níveis de albumina sérica em pacientes com CVC em estágios mais avançados da DRC (36.61 ± 4.37 g/L, p<0.05).

O único marcador inflamatório associado à CVC foi a proteína C reativa (PCR).^12 – 14 , 18^ Guerraty et al.^12^ demonstraram correlação direta entre PCR e gravidade da Calcificação da Válvula Aórtica (CVA), considerando níveis 4.85 ± 7.29 mg/L para CVA moderada e 5.48 ± 8.77 mg/L para CVA grave, p=0,01. Rong et al.^13^ observaram que pacientes com qualquer CVC possuíam níveis superiores da PCR (valor médio de 5.5, de 0.5-16.35 mg/L, p=0,04.). Já dentre os estudos que abarcaram pacientes sob TRS, Avila-Díaz et al.^14^ obtiveram associação com RR=1.09, IC95% 1.04-1.19, p=0,04, enquanto Plytzanopoulou et al.^18^ relataram AUC = 0,692, IC95% 0,58–0,89, p=0,03.

As alterações em fatores minerais e hormonais apareceram com frequência nos estudos incluídos. Níveis séricos de fosfato,^3 , 10 , 11 , 15 , 23^ cálcio,^17 , 21 , 25 – 27^ produto CaxP,^11 , 15^ PTH,^14 , 16 , 25 , 26^ FGF-23^23 , 25 , 26^ e do cofator Klotho^23 , 25^ foram associados direta e positivamente a CVC.

## Discussão

Foram identificados diversos preditores de calcificação valvar e valvopatias em pacientes com DRC, sendo a idade avançada o mais citado, com valores de corte superiores a 55 anos. Os resultados foram similares em estudos que calcularam a chance de CVM e CVA separadamente, permitindo concluir que o envelhecimento afeta similarmente ambas as válvulas, corroborando com outros estudos.^28 , 29^ Mesmo em pacientes sem DRC, a idade é relevante para o desenvolvimento de calcificação valvar de etiologia degenerativa, com prevalência em 10% da população idosa,^30^ além de ser um fator de risco tradicional para outras condições cardiovasculares.^9^

A redução progressiva e irreversível da TFG, persistente por ao menos três meses, e em níveis inferiores a 60 mL/min/1,73m^2^,^2^ constituiu um dos critérios para o diagnóstico de DRC.^4^ Nesta revisão sistemática, os estudos mostraram associação entre TFG e CVC a partir de 45-50ml/min/1,73m^2^.^10 , 12 , 20^ A duração da TRS e hipoalbuminemia também foram identificados como fatores de risco. A maioria dos estudos investigou a possibilidade de associação da cronicidade da HD com CVC, ^16 , 19 , 21 , 24^ e a duração variou entre 19,6 meses e 13,4 anos. London et al.^31^ afirmaram que os mecanismos de diálise promovem um ciclo repetitivo de estresse valvar devido à velocidade do fluxo sanguíneo e o grau de turbulência. Em relação à comparação entre HD e, Wang et al.^29^ afirmaram que condições cardiovasculares são similares entre os pacientes de ambas as modalidades. Em contrapartida, Mary Laxton,^32^ em artigo de revisão, apontou que a DP pode lentificar o processo de calcificação por preservar certa função renal residual, superando a HD. Não é possível afirmar que a maior frequência de achados em relação à HD, nesta presente revisão sistemática, deva-se a um maior risco de tal modalidade, uma vez que aproximadamente 90% da população sob alguma TRS a realiza.^33^

Esta revisão sistemática observou associação entre baixos níveis séricos de albumina (< 3,5 g/d) e CVC em pacientes sob TRS. Santos et al.^34^ concluíram que, além da desnutrição, fatores como redução na taxa de síntese devido a acidose metabólica, inflamação e insuficiência na ingestão proteica, a hemodiluição e a membrana utilizada na TRS (principalmente quando esterilizadas com hipoclorito de sódio) favorecem essa redução de albumina sérica em pacientes sob HD.^34^ Disfunção endotelial e aterosclerose secundárias à ação das proteínas de fase aguda e de citocinas inflamatórias também poderiam explicar a CVC.^9^ Vale ressaltar que Wang et al.^35^ observaram que pacientes com CVC sob DP eram significantemente mais desnutridos e tinham menores níveis de albumina sérica do que aqueles sem CVC, e a prevalência de CVC era maior quanto menor fossem tais níveis. Extrapolando a CVC, hipoalbuminemia é um conhecido preditor independente de eventos cardiovasculares de forma geral e de mortalidade e inflamação sistêmica crônica na DRC.^9^

A evidência científica atual aponta para uma relação positiva entre a elevação de marcadores inflamatórios e DRC, como observado por Shankar et al.^36^ que avaliou os fatores IL-6, TNF- α, PCR e contagem das células brancas, com maior associação para o TNF- α. Nesta revisão sistemática, três marcadores foram associados à CVC: a PCR, IL-6 e TNF- α, sendo apenas a primeira citada também em pacientes sob TRS. É sabido que a inflamação é comum nos estágios mais terminais da DRC e que o processo de calcificação tem origem principalmente em áreas sob inflamação crônica, como observado na gênese de placas ateroscleróticas.

O hiperparatireoidismo secundário à DRC parece estar relacionado ao desenvolvimento de CVC. A hiperfosfatemia decorrente da incapacidade de excreção está associada à calcificação de ambas as válvulas mitral e aórtica.^37^ Hipercalcemia e níveis elevados de FGF-23 também já foram estudados em pacientes com DRC. Nesta revisão sistemática, esses indicadores se associaram a CVC. Na DRC, a perda de néfrons prejudica a capacidade de o rim excretar fosfatos, levando à formação aumentada de complexos de cálcio-fósforo. A redução resultante dos níveis de cálcio ionizado estimula os receptores sensíveis ao cálcio existentes nas glândulas paratireoides a secretarem PTH a fim de aumentar a excreção renal destes componentes. Entretanto, o PTH aumenta o cálcio sérico ao estimular a reabsorção a partir dos ossos e dos rins. Concomitantemente, a conversão da vitamina D ao calcitriol ainda aumenta a reabsorção intestinal de ambos os íons. Tais desordens culminam no depósito extracelular de cálcio.

Esta revisão sistemática possui algumas limitações: alguns dos estudos incluídos possuíam pequenas amostras e curto tempo de seguimento. Apenas estudos de metodologia observacional foram englobados, sendo a maioria deles de corte transversal, que limita as inferências de causalidade. Os estudos também apresentaram variações em populações analisadas, grupos de comparação, critérios de inclusão e exclusão, bem como nas formas de mensuração dos resultados, escores e técnicas estatísticas divergentes entre si, o que dificulta uma adequada comparação dos resultados.

Esta heterogeneidade constitui uma limitação, visto que compromete a comparabilidade e validade externa dos achados. Entretanto tal fator não invalida os achados da revisão sistemática. A heterogeneidade dos achados obtidos pode ser explorada por meio de análises estatísticas apropriadas, como modelos de efeitos aleatórios em meta-análises, observando-se uma oportunidade para novas investigações e fornecendo direções para futuras pesquisas. Diferentes metodologias oferecem perspectivas complementares, enriquecendo a compreensão do tema, de forma que dezenas de fatores de risco foram apontados pelos estudos incluídos. O acompanhamento regular e a vigilância desses parâmetros na prática clínica podem propiciar a identificação precoce dos pacientes com DRC sob risco de calcificação valvar e valvopatias, possibilitando melhores oportunidades de prevenção e tratamento, com consequente aumento de sobrevida e qualidade de vida.

## Conclusão

Neste estudo, foram identificados diversos fatores de risco para o desenvolvimento de calcificação valvar em pacientes com DRC, sendo os principais a idade, estágios avançados da doença, duração da TRS, hipoalbuminemia, citocinas e outros agentes inflamatórios, além de produtos envolvidos no metabolismo do hiperparatireoidismo secundário.

Disponibilidade de Dados

Os conteúdos subjacentes ao texto da pesquisa estão contidos no manuscrito.

## References

[1] Marwick TH, Amann K, Bangalore S, Cavalcante JL, Charytan DM, Craig JC (2019). Conference Participants. Chronic Kidney Disease and Valvular Heart Disease: Conclusions from a Kidney Disease: Improving Global Outcomes (KDIGO) Controversies Conference. Kidney Int.

[2] Foley RN, Parfrey PS, Sarnak MJ (1998). Clinical Epidemiology of Cardiovascular Disease in Chronic Renal Disease. Am J Kidney Dis.

[3] Tian Y, Feng S, Zhan Z, Lu Y, Wang Y, Jiang S (2016). Risk Factors for New-Onset Cardiac Valve Calcification in Patients on Maintenance Peritoneal Dialysis. Cardiorenal Med.

[4] Goldman ADC (2014). Goldman Cecil: Medicina.

[5] Umana E, Ahmed W, Alpert MA (2003). Valvular and Perivalvular Abnormalities in End-Stage Renal Disease. Am J Med Sci.

[6] Rostand SG, Brunzell JD, Cannon RO, Victor RG (1991). Cardiovascular Complications in Renal Failure. J Am Soc Nephrol.

[7] Usuku H, Yamamoto E, Oike F, Tsunoda R, Nishigami K, Sakaguchi T (2019). Development of Caseous Calcification of Mitral Annulus after Initiation of Hemodialysis Therapy. J Cardiol Cases.

[8] Wang C, Jiang L, Feng S, Shi Y, Shen H, Shi X (2013). Risk Factor Analysis of Calcification in Aortic and Mitral Valves in Maintenance Peritoneal Dialysis Patients. Kidney Blood Press Res.

[9] Querido S, Quadros Branco P, Sousa HS, Adragão T, Gonçalves PA, Gaspar MA (2017). Hypervolemia, Hypoalbuminemia and Mitral Calcification as Markers of Cardiovascular Risk in Peritoneal Dialysis Patients. Rev Port Cardiol.

[10] Alamir M, Radulescu V, Goyfman M, Mohler ER, Gao YL, Budoff MJ (2015). Prevalence and Correlates of Mitral Annular Calcification in Adults with Chronic Kidney Disease: Results from CRIC Study. Atherosclerosis.

[11] Fernández LM, Sánchez-Alvarez JE, de la Tassa CM, Fernández JJB, María V, Fernández E (2021). Risk Factors Associated with Valvular Calcification in Patients with Chronic Kidney Disease. Analysis of NEFRONA study. Nefrologia.

[12] Guerraty MA, Chai B, Hsu JY, Ojo AO, Gao Y, Yang W (2015). Relation of Aortic Valve Calcium to Chronic Kidney Disease (from the Chronic Renal Insufficiency Cohort Study). Am J Cardiol.

[13] Rong S, Qiu X, Jin X, Shang M, Huang Y, Tang Z (2018). Risk Factors for Heart Valve Calcification in Chronic Kidney Disease. Medicine.

[14] Avila-Díaz M, Mora-Villalpando C, Prado-Uribe Mdel C, Orihuela-Rodriguez O, Villegas-Antelo E, Gómez-Noriega AM (2013). De Novo Development of Heart Valve Calcification in Incident Peritoneal Dialysis Patients. Arch Med Res.

[15] Engole YM, Lubenga YN, Nlandu YM, R Makulo JR, Mokoli VM, Kahindo CK (2020). Prevalence, Location, and Determinants of Valvular Calcifications in Congolese Patients on Chronic Hemodialysis: A Multicenter Cross-Sectional Study. Saudi J Kidney Dis Transpl.

[16] Guo J, Zeng M, Zhang Y, Huang H, Yang G, Xu F (2020). Serum Alkaline Phosphatase Level Predicts Cardiac Valve Calcification in Maintenance Hemodialysis Patients. Blood Purif.

[17] Ikee R, Honda K, Ishioka K, Oka M, Maesato K, Moriya H (2010). Differences in Associated Factors Between Aortic and Mitral Valve Calcification in Hemodialysis. Hypertens Res.

[18] Plytzanopoulou P, Papasotiriou M, Politis P, Mavrikakis M, Karagiannis G, Tzavella E (2020). Malnutrition as a Risk Factor for Cardiac Valve Calcification in Patients Under Maintenance Dialysis: A Cross-Sectional Study. Int Urol Nephrol.

[19] Sayarlioglu H, Acar G, Sahin M, Altunoren O, Yavuz YC, Nacar AB (2013). Prevalence and Risk Factors of Valvular Calcification in Hemodialysis Patients. Iran J Kidney Dis.

[20] Asselbergs FW, Mozaffarian D, Katz R, Kestenbaum B, Fried LF, Gottdiener JS (2009). Association of Renal Function with Cardiac Calcifications in Older Adults: The Cardiovascular Health Study. Nephrol Dial Transplant.

[21] Xiong JQ, Chen XM, Liang CT, Guo W, Wu BL, Du XG (2022). Prognosis and Risk Factors for Cardiac Valve Calcification in Chinese End-Stage Kidney Disease Patients on Combination Therapy with Hemodialysis and Hemodiafiltration. Ren Fail.

[22] Fox CS, Larson MG, Vasan RS, Guo CY, Parise H, Levy D (2006). Cross-Sectional Association of Kidney Function with Valvular and Annular Calcification: The Framingham Heart Study. J Am Soc Nephrol.

[23] Silva AP, Viegas CSB, Guilherme P, Tavares N, Dias C, Rato F (2022). Gla-Rich Protein, Magnesium and Phosphate Associate with Mitral and Aortic Valves Calcification in Diabetic Patients with Moderate CKD. Diagnostics.

[24] Usuku H, Yamamoto E, Arima Y, Kaikita K, Matsui H, Tsujita K (2019). Accumulation of Coronary Risk Factors is Associated with Progression of Mitral Annular Calcification in Patients Undergoing Dialysis Therapy: A Long-Term Follow-Up Study. Int J Cardiol.

[25] Chen Y, Chen YX, Huang C, Duan ZB, Xu CY (2021). The Clinical Value of Klotho and FGF23 in Cardiac Valve Calcification among Patients with Chronic Kidney Disease. Int J Gen Med.

[26] Di Lullo L, Gorini A, Bellasi A, Morrone LF, Rivera R, Russo L (2015). Fibroblast Growth Factor 23 and Parathyroid Hormone Predict Extent of Aortic Valve Calcifications in Patients with Mild to Moderate Chronic Kidney Disease. Clin Kidney J.

[27] Hoshina M, Wada H, Sakakura K, Kubo N, Ikeda N, Sugawara Y (2012). Determinants of Progression of Aortic Valve Stenosis and Outcome of Adverse Events in Hemodialysis Patients. J Cardiol.

[28] Strózecki P, Odrowaz-Sypniewska G, Manitius J (2005). Cardiac Valve Calcifications and Left Ventricular Hypertrophy in Hemodialysis Patients. Ren Fail.

[29] Wang AY, Wang M, Woo J, Lam CW, Li PK, Lui SF (2003). Cardiac Valve Calcification as an Important Predictor for All-Cause Mortality and Cardiovascular Mortality in Long-Term Peritoneal Dialysis Patients: A Prospective Study. J Am Soc Nephrol.

[30] Tarasoutchi F, Montera MW, Ramos AIO, Sampaio RO, Rosa VEE, Accorsi TAD (2020). Update of the Brazilian Guidelines for Valvular Heart Disease - 2020. Arq Bras Cardiol.

[31] London GM, Pannier B, Marchais SJ, Guerin AP (2000). Calcification of the Aortic Valve in the Dialyzed Patient. J Am Soc Nephrol.

[32] Laxton MK (2016). Peritoneal Dialysis: An Effective Yet Underused Renal Replacement Therapy. JAAPA.

[33] Marinho AWGB, Penha AP, Silva MT, Galvão TF (2017). Prevalência de Doença Renal Crônica em Adultos no Brasil: Revisão Sistemática da Literatura. Cad Saúde Coletiva.

[34] Santos NSJ, Draibe SA, Kamimura MA, Cuppari L (2004). Albumina Sérica como Marcador Nutricional de Pacientes em Hemodiálise. Rev Nutr.

[35] Wang AYM, Woo J, Wang M, Sea MMM, Ip R, Li PKT (2001). Association of Inflammation and Malnutrition with Cardiac Valve Calcification in Continuous Ambulatory Peritoneal Dialysis Patients. J Am Soc Nephrol.

[36] Shankar A, Sun L, Klein BE, Lee KE, Muntner P, Nieto FJ (2011). Markers of Inflammation Predict The Long-Term Risk of Developing Chronic Kidney Disease: A Population-Based Cohort Study. Kidney Int.

[37] Ureña-Torres P, D’Marco L, Raggi P, García-Moll X, Brandenburg V, Mazzaferro S (2020). Valvular Heart Disease and Calcification in CKD: More Common than Appreciated. Nephrol Dial Transplant.

[38] Genctoy G, Eldem O, Ergun T, Arikan S (2015). Periaortic Fat Tissue: A Predictor of Cardiac Valvular Calcification, Malnutrition, Inflammation, and Atherosclerosis Components in Hemodialysis Patients. International Center for Artificial Organs and Transplantation and Wiley Periodicals.

