# Supplementary material for: Preditores Clínicos e Laboratoriais do Desenvolvimento de Valvopatias na Doença Renal Crônica: Uma Revisão Sistemática
Source: Arq Bras Cardiol. 2025 Aug 14;122(8):e20240222. [Article in Portuguese] doi: 10.36660/abc.20240222 (PMC12671838; doi:10.36660/abc.20240222)
Supplement: Translation

# Translation: Clinical and Laboratory Predictors for the Development of Heart Valve Diseases in Chronic Kidney Disease: A Systematic Review

**DOI:** 10.36660/abc.20240222i

**Authors:** Hayala Machado Cavalcante Conceição, Ana Luísa Vaz Valois, Erick Magalhães Silva Ramos, Ana Marice Ladeia

**Affiliations:** 1 Salvador BA Brazil Escola Bahiana de Medicina e Saúde Pública, Salvador, BA – Brazil; 2 Salvador BA Brazil Universidade Salvador, Salvador, BA – Brazil

**Keywords:** Heart Valve Diseases, Chronic Renal Insufficiency, Risk Factors

## Abstract

**Background:**

Chronic kidney disease (CKD) is associated with a higher prevalence of valvular diseases and increased mortality from cardiovascular causes. Factors that influence the genesis of cardiac valve calcification (CVC) in these patients are not well-defined.

**Objective:**

To determine the risk factors for valvular calcification in patients with CKD.

**Methods:**

Systematic review based on PRISMA, which included observational studies evaluating the association of clinical and laboratory data with CVC in patients with CKD, undergoing or not hemodialysis or peritoneal dialysis. Articles were retrieved from databases (MEDLINE; SCIELO; CENTRAL; EMBASE; LILACS/BVS) and selected blindly by two authors; discrepancies were resolved by a third author. Data collection and synthesis were carried out by the main author. The assessment of methodological quality and risk of bias was based on STROBE and Newcastle-Ottawa guidelines.

**Results:**

A total of 783 studies were identified, of which 20 were included, encompassing 13,314 patients from 10 countries. The factors most strongly associated with CVC were age >55 years, glomerular filtration rate <53 mL/min/1.73m^2^, renal replacement therapy (RRT) >20 months, hypoalbuminemia, C-reactive protein (CRP), serum levels of IL-6, TNF-α, parathyroid hormone, hyperphosphatemia, hypercalcemia, Ca × P product, and FGF-23 resulting from secondary hyperparathyroidism. Both mitral and aortic valves were studied, and no differences were observed between hemodialysis and peritoneal dialysis.

**Conclusion:**

Age, RRT, chronic inflammation, and secondary hyperparathyroidism promote calcium and phosphate deposition in the valves, making CKD patients more susceptible to CVC) Monitoring these parameters provides opportunities for prevention and treatment.

## Introduction

Cardiovascular causes account for more than 50% of deaths in patients with chronic kidney disease (CKD) compared to the general population,^1^and the prevalence of valvular heart diseases is significantly higher in this group.^2^Valvular abnormalities, mainly in the aortic and mitral valves, as well as the need for hemodialysis (HD) or peritoneal dialysis (PD), are markers of morbidity and mortality. However, this is one of the least addressed aspects in the study of CKD, with its relevance often overlooked by many professionals.

The pathogenesis of valvular heart diseases involves inflammatory processes (both systemic and local), modified action of parathyroid hormone (PTH) in secondary hyperparathyroidism,^3 – 5^disorders in calcium-phosphorus metabolism,^5 , 6^the type of dialysis (HD or PD), duration of dialysis,^3 , 7 , 8^serum cholesterol levels,^3 , 9^albumin levels,^3 , 9^and blood volume.^9^However, the aspects truly involved in the pathogenesis of valvular calcification and valvular diseases in patients with CKD, which justify the higher prevalence of such conditions in this group, are not well defined.^2^

The objective of this systematic review is to identify possible clinical and laboratory predictors that constitute risk factors for valvular calcification in individuals with CKD, undergoing or not HD or PD.

## Methods

This systematic review was conducted according to the Preferred Reporting Items for Systematic Reviews and Meta-Analysis protocols (PRISMA-P) and submitted for registration in the Prospective Register of Systematic Reviews (PROSPERO) (CRD42021291576).

### Search strategy

The search strategy used DeCS (Health Sciences Descriptors) and MeSH Terms (Medical Subject Headings) on MEDLINE (Medical Literature Analysis and Retrieval System), LILACS (Latin American and Caribbean Health Sciences Literature), SCIELO (Scientific Electronic Library Online), Cochrane Library (CENTRAL), and EMBASE platforms. The MeSH Terms search was as follows: ((((((End-Stage Kidney Disease) OR (Chronic Kidney Failure) OR (End-Stage Renal Disease) OR (Chronic Renal Failure) OR (End-Stage Renal Failure)))))) OR (Dialysis) AND ((Risk Factor) OR (Risk Factors)) AND ((Heart Valve Disease) OR (Valvulopathy)) AND (Calcification). The search of DeCS terms was as follows: (Insuficiência Renal Crônica) OR (Doença Renal Crônica) AND (Valvulopatia) OR (Valvopatia).

### Eligibility criteria

Observational studies in Portuguese, English, and Spanish that included patients with CKD (with or without dialysis) and assessed the association between clinical/laboratory parameters and valvular calcification were considered eligible. Studies with incomplete data or those that did not meet all the criteria were excluded.

### Identification and selection of studies

Two authors selected the articles using the Rayyan QCRI software, and disagreements were resolved by a third author. The Kappa Concordance Test (K) assessed agreement between reviewers. References from relevant articles were checked, and the selection steps were summarized in PRISMA flowchart.

### Data extraction and analysis

Data collection was performed by the main author using an electronic form based on the Critical Appraisal and Data Extraction for Systematic Reviews of Prognostic Factor Studies (CHARMS-PF Checklist).

The methodological quality and risk of bias were assessed using The Strengthening the Reporting of Observational Studies in Epidemiology (STROBE) and the Newcastle-Ottawa Scale (NOS), respectively. The studies included showed moderate performance in the analyses, as shown in the tables below.

## Results

### Study selection

A total of 790 articles were retrieved; 783 from databases and seven through manual search and reference lists. After removing duplicates and applying eligibility criteria, 20 articles were selected ( Figure 1 ), including 13 cross-sectional studies, six cohort studies, and one case-control study, encompassing 13,314 patients from 10 countries, with sample sizes ranging from 30 to 3,929 participants. The agreement between examiners in the article selection process showed a Kappa value > 0.758 (good agreement).

**Figure 1:** Flowchart of the studies evaluated in this review.

Some studies evaluated patients with CKD without categorizing the stages of the disease (which range from 1 to 5 according to the literature),^4^while others included only patients in specific stages. There were studies that included patients undergoing renal replacement therapy (RRT) and others that specifically evaluated HD or PD. The included articles employed multivariate analyses and adopted distinct methodologies.

Table 1 summarizes the main characteristics of the 20 included studies, with information on population samples and scores obtained by each of them.

**Table 1:** General characteristics of the studies included in the systematic review, population samples, and scoring in quality analysis

Authors, year, country	Study design	Sample description (n)	Endpoint	Male sex (%)		Mean age (years)		STROBE	Newcastle-Ottawa
Alamir et al., 2015, USA^10^	Cross-sectional	Mild/moderate CKD (n=2070)	MAC; noncontrast CT	53.7%		58 (21-74)		86.36%	4
Asselbergs et al., 2008, USA^20^	Retrospective cohort	DRC (n=3929)	MAC, AAC, AAS; 2D-ECHO	60%		74 ± 5		90.9%	5
Àvila-Díaz et al., 2013, Mexico^14^	Prospective cohort	CKD under PD (CAPD ou DPA) (n=124)	CVC; 2D-ECHO	68.5%		46 (30-54)		95.45%	7
Chen et al., 2021, China^25^	Case-control	CKD stages 2 - 5 (n=180)	CVC; 2D-ECHO	63%		58 ± 14		86.36%	2
Di Lullo et al., 2015, Italy^26^	Cross-sectional	CKD stages 3 - 4 (n=100)	CVC; 2D-ECHO	60%		51 (46-56)		72.72%	4
Engole et al., 2020, Democratic Republic of the Congo^15^	Cross-sectional	CKD under HD (n=60)	CVC; 2D-ECHO	71.6%		52.5 ± 15.9		72.72%	6
Fernandez et al., 2021, Spain^11^	Prospective cohort	CKD stages 2- 5, under RRT or not (n=397)	CVC; 2D-ECHO	61%		59.1 ± 11.5		72.72%	5
Fox et al., 2006, USA^22^	Cross-sectional	DRC (n=3047)	CVC; 2D-ECHO	47.6%		59 ± 10		90.9%	5
Genctoy et al., 2015, Turkey^38^	Cross-sectional	CKD under HD (n=76)	CVC; 2D-ECHO	64.47%		60.5 ± 15.5		77.27%	6
Guerraty et al., 2015, USA^12^	Cross-sectional	TFG 20-70 ml/min/1,73m^2^e CVA (n=1923)	CVA; noncontrast CT	53%		58.45 ± 11.45		90.9%	7
Guo et al., 2020, China^16^	Cross-sectional	CKD under HD (n=145)	CVC; 2D-ECHO with Doppler	54.5%		50 (23-74)		81.8	5
Hoshina et al., 2011, Japan^27^	Retrospective cohort	CKD under HD (n=30)	AS; 2D-ECHO	Rapid progression of AS (n=30): 40%	Slow progression of AS (n=30): 53%	Rapid progression of AS: 73.6 ± 6.1	Slow progression of AS: 69.8 ± 7.5	81.81%	6
Ikee et al., 2010, Japan^17^	Cross-sectional	CKD under HD (n=112)	CVC; 2D-ECHO	68.7%		67 ± 10		86.36%	6
Plytzanopoulou et al., 2020, Grécia^18^	Cross-sectional	CKD under HD (n=42)	CVC; 2D-ECHO	69%		72.97 ± 11.6		95.45%	5
Rong et al., 2018, China^13^	Cross-sectional	DRC (n=288)	CVC; 2D-ECHO	65.9%		CVC: 70.42±11.83;	Without CVC: 56.47±15.00	81.81%	3
Sayarlioglu et al., 2013, Turkey^19^	Cross-sectional	CKD under HD (n-129)	CVC; 2D-ECHO	CVC: 52.3%	Sem CVC: 46.5%	CVC: 48. 2 ± 16.8	Without CVC: 60.3 ± 13.5	77.27%	6
Silva et al., 2022, Portugal^23^	Cross-sectional	Diabetic patients with CKD stages 2- 4 (n=80)	CVC; 2D-ECHO	71.3%		56 ± 8.1		81.81%	6
Tian et al., 2016, China^3^	Prospective cohort	DRC sob DP (n=194)	CVC; 2D-ECHO	54%		61.6 ± 12.5		90.9%	7
Usuku et al., 2019, Japan^24^	Retrospective cohort	CKD under PD (n=95)	MAC; 2D-ECHO	CAM (n=28): 54%	Without CAM (n=67): 54%	CAM (n=28): 65 ± 10.7	Without CAM (n=67): 62.6 ± 13.2	86.36%	7
Xiong et al., 2022, China^21^	Cross-sectional	End-stage CKD, under HD (n=293)	CVC; 2D-ECHO, CT and/or CTA in some cases	54.6%		64 ± 7.0		77.27%	4

CTA: computed tomography angiography; MAC: mitral annulus calcification; AAC: aortic annulus calcification; CVC: cardiac valve calcification; PD: peritoneal dialysis; CKD: chronic kidney disease; AS: aortic stenosis; SLAS: slow progression aortic stenosis; AAS: aortic annulus sclerosis; 2D-ECHO: two-dimensional echocardiography; HD: hemodialysis; CT: computed tomography; RRT: renal replacement therapy; the statistical significance level was p < 0.05, except for the study by Engole et al.,^15^which did not specify the p-value.

### Summary of results

A total of 38 risk factors for valvular calcification were identified. Table 2 summarizes studies that evaluated risk factors in patients with CKD in general. Table 3 describes the results of studies that included only patients undergoing RRT, whether HD or PD. The findings are compiled in the Central Figure.

**Table 2:** Risk factors identified in studies that included patients with chronic kidney disease

Authors, year, country	Endpoints and methods of definition	Risk factors identified in the study	Valve calcification and/or heart valve diseases identified
Alamir et al, 2015, USA^10^	MAC in two CTs by the Agatston algorithm	MAC associated with GFR < 50mL/min/1.73m^2^ (ORa = 2.30, 95%CI 1.40-3.79), age > 55 years (OR = 4.01, 95%CI 2.55-6.32), phosphate > 4.1 mg/dL (OR = 3.33, 95%CI 1.37-8.09)	Previous MVC: 331 (16%).
Asselbergs et al, 2008, USA^20^	MAC and AAC; hyperechogenicity in 2D-ECHO AAS; aortic cuspid thickening and flpw velocity <2.0m/s	TFG <45 mL/min/1,73 m2 (OR =1.15, 95%IC 1.03-1.12, p=0,015) and cystatin C (OR = 1.12; 95%CI 1.03-1.23, p=0,013) associated with previous CAM	Mild ACM: 1495, moderate MAC: 133, severe MAC: 12. AAC: mild: 1698, moderate: 49, severe: 1. Aortic sclerosis in 2114.
Chen et al., 2021, China^25^	CVC hyperechogenic masses with a diameter ≥1 mm in 2D-ECHO Doppler	CVC was associated with serum creatinine (CKD stage 5, 845.09 ± 334.18 μmol/L, p<0.05), FGF-23 (CKD stage 5, 1039.43 ± 214.83 pg/ml, p<0.05), and Klotho protein (CKD stages 2-3, 159.05 ± 27.53 U/L, p<0.05). Patients in stage 5 had higher levels of Hb (90.52 ± 23.36 g/L, p<0.05), albumin (36.61 ± 4.37 g/L, p<0.05), osteocalcin (197.32 ± 78.88 ng/ml, p<0.05), PTH (392.40 ± 233.88 pg/ml, p=0.05), and bone-specific ALP (86.36 ± 18.18 U/L, p=0.05), along with lower corrected serum calcium levels (2.06 ± 0.15 mmol/L, p<0.05)	Total CVCl: 41 (22,7%); 4 patients with CKD 2-3; 12 patients with CKD 4, 25 patients with CKD 5
Di Lullo et al., 2015, Italy^26^	CVC by Wilkins score in 2D-ECHO	AVC and PTH (r2 = 0,212 p=0,03) and FGF-23 (r2 = 0,272; p=0.01) MVC score associated with serum calcium (r2 = 0,565; p=0.01)	CVC total: 100%. MVC: 96; 61 had score 1; 34 had score 2, 1 had score 3 AVC: 100 (100%)
Fernandez et al., 2021, Spain^11^	MVC masses > 5mm and AVC > 2mm, in 2D-ECHO	MVC associated with age (OR = 1.05. 95%CI 1.03-1.08), peripheral vascular disease (OR = 4.22, 95%CI 1.53-13.12) and Ca x P product (OR = 1.03, 95%CI 1.01-1.05). AVC associated with age (OR = 1.10, 95%CI1.05-1.15), P levels (OR = 1.64, 95%CI 1.16-2.42), atherosclerosis and greater carotid plaque area (OR = 3.10, 95%IC 1.18-8.80).	MVC increased from 96 (24.2%) to 123 (31%). AVC increased from 119 (30%) to 171 (43.1%).
Fox et al., 2006, USA^22^	CVC; MVC hyperechogenic masses > 0.3cm in M-mode or > 1/3 of annulus in the parasternal window AVC if > ½ of hyperechogenic annulus	CVC associated with SAH (67%, p<0,01), DM (21%, p<0,05), total cholesterol/HDL (4.8 ± 1.8; p<0.01) and BMI (29.2 ± 5.2 Kg/^2^; p<0.001); GFR significantly lower in patients with CVC (GFR = 78 ± 25mL/min/1.73m^2^, p<0.001)	CVC total: 284 (9,3%). MVC: 130 (4,3%). AVC: 112 (3,7%). AAS: 188 (6,2%).
Guerraty et al., 2015, USA^12^	AVC by Agatston score, noncontrast CT	Independent association with moderate/severe AVC and age (62.26 ± 7.9 and 66.53 ± 7; p<0.001, respectively); BMI: 31.73 and 31.72 (p<0.0007); AC (105.01 ± 15.53 and 106.81 ± 14.31; p<0.001); SBP (127.75 ± 21.99 and 132.28 ± 21.21; p<0.001); hypercholesterolemia (463/90% and 401/94%; p<0.001); HbA1c (6.73 ± 1.52% and 6.64 ± 1.36%; p<0.001); reduced MET (198.37 ± 25.59 and 178.24 ± 120.95; p<0.001; DM (281/55% and 52/59%; p<0.001); SAH (478/93% and 409/96%; p<0.001; cardiovascular disease (149/29% and 176/41%; p<0.001); CRP (4.85 ± 7.29 and 5.48 ± 8.77; p=0.0100); total plasma homocysteine (14.17 ± 4.86 and 16.55 ± 7.06; p<0.0001); ALP (100.84 ± 34.99 and 99.7 ± 34.23; p=0.0041). Mean GFR was 44.6 mL/min/1.73m^2^and was associated with AVC (p<0.0001).	mild/moderate AVC: 515 (26.8%) Severe AVC: 426 (22.1%).
Rong et al, 2018, China^13^	CVC total, hyperechogenic masses > 1mm in 2D-ECHO with Doppler	CVC was associated with age (70.42 ± 11.83 years, p<0.001; OR = 1.091, 95% CI 1.048–1.136, p<0.05), lower levels of pre-albumin (238.44 ± 91.48 g/L, p=0.05), cholesterol (OR = 0.488, 95% CI 0.306–0.780, p=0.03), triglycerides (1.4 ± 0.65 mmol/L, p=0.37), and APO-E (37.3, ranging from 30.2–45.6 mg/L, p=0.09), LDL (OR = 163.028, 95% CI 3.796–7002.467, p=0.08), CRP (5.5, ranging from 0.5–16.35 mg/L, p=0.04), and IL-6 (18.76, ranging from 5.95–46.9 pg/mL, p=0.05). The group with CVC at more advanced stages of CKD (stage 5 under dialysis) showed a prevalence of 30.3% with CVC compared to 18.9% without CVC, p=0.048.	CVC total: 66 (22,9%). MVC: 14 (21.2% of 66); AVC: 100% of 66)
Silva et al,, 2022, Portugal^23^	CVC total; MVC by Wilkins score and AVC according to the score proposed by Lullo et al, in 2D-ECHO	MVC e AVC were associated with lower eGFR (p< 0.0001), GRP (p< 0.0001), Mg (p= 0.029 for MVC and p= 0.001 for AVC), and α-Klotho (p= 0.002), as well as higher levels of P (p= 0.001), PTH (p= 0.025 for MVC and p=0.030 for AVC), FGF-23 (p< 0.0001), and TNF-α (p=0.037). There was a negative correlation between GRP and MVC (r = −0.754, p< 0.0001), and low levels of GRP were identified as an independent risk factor for MVC (ORa = 0.268, 95% CI 0.101–0.725, p= 0.005; aPR = 0.750; 95% CI 0.456–0.976; p= 0.024) and AVC (ORa = 0.202, 95% CI 0.109–0.401, p= 0.022; aPR = 0.813; 95% CI 0.113–0.937; p< 0.0001). Similarly, low levels of Mg were risk factors for MVC (ORa = 0.747, 95% CI 0.263–0.921, p= 0.003; aPR = 0.762; 95% CI 0.256–0.963; p= 0.02) and AVC (ORa = 0.580, 95% CI 0.173–0.948, p= 0.008; aPR = 0.809; 95% CI 0.391 0.974; p= 0.006). High levels of P were also identified as risk factors (for MVC, ORa = 1.078, 95% CI 1.0–1.612, p= 0.001; for AVC, ORa = 1.497, 95% CI 1.004–2.378, p= 0.002) and FGF-23 (for MVC, ORa = 1.209, 95% CI 1.099–1.619, p= 0.035; for AVC, ORa = 1.126, 95% CI 1.034–1.436, p= 0.011).	CVM: 29 (36.2%); AVC: 29 (36.2%).

AAC: aortic annulus calcification; ALP: alkaline phosphatase; APO-E: apolipoprotein E; aPR: adjusted prevalence ratios; BMI: body mass index; CAPD: continuous ambulatory peritoneal dialysis; CA: abdominal circumference; MAC: mitral annulus calcification; CVC: cardiac valve calcification; AVC: aortic valve calcification; MVC: mitral valve calcification; DM: diabetes mellitus; CKD: chronic kidney disease; APD: automated peritoneal dialysis; 2D-ECHO: two-dimensional echocardiography; AAS: aortic annulus sclerosis; FGF-23: fibroblast growth factor 23; GRP: Gla-rich protein; SAH:: systemic arterial hypertension; Hb: hemoglobin; HbA1c: glycated hemoglobin; HDL: high density lipoprotein; ABI: ankle-brachial index; LDL: low density lipoprotein; MET: metabolic equivalent of task; Mg: magnesium; OR: odds ratio; ORa: adjusted odds ratio; P: phosphorus; SBP: systolic blood pressure; CRP: C-reactive protein; PTH: parathyroid hormone; CT: computed tomography; GFR: glomerular filtration rate; TG: triglycerides; TNF-α: tumor necrosis factor-alpha; RRT: renal replacement therapy; the level of significance was set at p<0.05.

**Table 3:** Risk factors identified in studies that included only chronic kidney disease patients under renal replacement therapy

Authors, year, country	Endpoints and methods of definition	Risk factors identified in the study	Valve calcification and/or heart valve diseases identified
Àvila-Díaz et al, 2013, Mexico^14^	CVC in general, luminous echo > 1 mm; 2D-ECHO	MVC associated with age (RR=1.051, 95%CI 1.06-1.09, p=0,02), DM (RR=0.287, 95%CI 0.1-0.8, p=0,01), osteoprotegerin (RR=1.15, 95%CI 1.03-1.21, p=0,008), PTH (RR=0.41, 95%CI 1.08-50.5, p=0,04) and PCR (RR=1.09, 95%CI 1.04-1.19, p=0,04). iPTH (RR = 2.002, 95%CI 1.052–3.81, p<0,034) for AVC	CVC total: 57 (46%); MVC: 15 (26.3%); AVC: 33 (57.8%); calcification in both valves: 9 (15.8%)
Engole et al., 2020, Democratic Republic of the Congo^15^	CVC in general, luminous echo > 1 mm; 2D-ECHO com Doppler.	Age > 60 years (aOR = 4.48; 95%CI 1.67-30.10, p=0.003), smoking (aOR=4.57; 95%CI 1.15-13.36, p=0.016), P levels (aOR=2.17; 95%CI 1.83-5.65, p=0.012) and hypertension (aOR=3.963, 95%CI 1.24–15.7, p=0.014) were independently associated with CVC	CVC total: 23 (38%); MVC: 23%; AVC: 64%
Genctoy et al., 2015, Turkey^38^	CVC in general, luminous echo > 1 mm; 2D-ECHO.	CVC associated with T-PAFT (OR=1.006, CI not presented; p=0,04)	CVC total: 50 (65,8%); 18 (23,7%) in one cuspid and 32 (42,1%) in two
Guo et al,, 2020, China^16^	CVC in general; calcification was assessed by GCCS; 2D-ECHO Doppler.	Age (r = 0.25; p=0.003), duration of hemodialysis (r = 0.27; p=0.001), serum iPTH (r = 0.18; p=0.03), and serum PA (r = 0.24; p=0.003; OR = 3.87, 95%CI 1.86–8.07, p<0.001; aOR = 3.92, 95% CI 1.37–11.2, p=0.011). In an analysis using cubic splines, the probability of GCCS = 1 significantly increased when PA exceeded 232 U/L. There was a negative correlation with serum albumin (r = −0.22; p=0.008). PA > 232 U/L and age > 60 years are determinants for high risk of CVC.	CVC GCCS ≥ 1: 83
Hoshina et al., 2011, Japan^27^	Aortic stenosis defined based on PPG progression between two 2D-ECHO within 3 months	Systolic blood pressure (161 ± 21.5 mmHg, OR = 1.06, 95%CI 1-1.11, p=0,04) and calcium serum levels (9.66 ± 1.05 md/dL, OR = 6.08, 95%CI 1.28-28.8, p=0,02).	Fast progression of AS: 15; rapid progression of AS: 15
Ikee et al., 2010, Japan^17^	CVC in general, luminous echo > 1 mm; 2D-ECHO	Age associated with CVC (for AVC, OR = 1.06, 95%CI 1.01-1.11, p=0,01). For MVC, OR = 1.04, 95%CI 1.01-1.09, p=0.04). Calcium associated with AVC (OR = 2.16, 95%CI 1.05-4.44, p=0.03) Serum B2-microglobulin was associated with MVC (OR = 1.10, 95%CI 1.01-1.2, p=0,01) and correlated with HD duration (r = 0.273; p=0.004), serum albumin (r = 0.209; p=0.02), total cholesterol (r = 0,243, p=0.01), triglycerides (r = 0.189, p=0.04), HDL-C (r = 0.337, p=0.001), and CRP (r = 0.246, p=0.009).	CVM: 58 (51,7%); AVC: 85 (75%); calcification in both valves: 48 (42,8%)
Plytzanopoulou et al., 2020, Greece^18^	CVC in general, 2D-ECHO.	Age (AUC 0.734, 95% CI 0.58–0.89, p=0.011), CRP (AUC 0.692, 95% CI 0.516–0.868, p=0.03), and decreased serum albumin (AUC 0.73, 95% CI 0.57–0.89, p=0.012) are positive predictors of CVC. Age >76.5 years (OR 9.56, 95% CI 1.54–59.42, p=0.015) and CRP >3.5 mg/dL (OR 9.26, 95% CI 1.51–56.83, p=0.016) are associated with severe CVC	Absent or mild CVC in 24 (57,15%); moderate or severe CVC in 18 (42,85%). MVC: 21 (50%); AVC: 16 (38,10%).
Sayarlioglu et al, 2013, Turkey^19^	CVC in general, 2D-ECHO.	Advanced age (60.3 ± 13.5 years, p<0.001) and low serum albumin (3.7 ± 0.5 mg/dL, p=0.02) were linked to calcification in both valves. DM was associated with MVC (16.3%, p=0.02). The duration of dialysis in patients with MVC and AVC was significantly longer compared to other groups (19.6 ± 40.6 months vs. 7.1 ± 5.8 months, p=0.01)	CVC total: 43 (33,3%); CVM: 30 (23,3%); CVA: 28 (21,7%).
Tian et al, 2016, China^3^	CVC in general, if luminous echo > 1 mm; 2D-ECHO	CVC associated with chronic PD (OR=1.039, 95%CI 1.004-1.075, p=0,03), serum P (OR=2.569, 95%CI 1.227-5.377, p=0,01), decreased serum albumin (OR albumin =0.935, 95%CI 0.877-0.997, p=0.01)	CVC: 97 (32%). MVC: 7 new cases; AVC: 30 new cases;
Usuku et al, 2019, Japan^24^	MAC defined by echo-dense mass ≥ 5mm in the atrioventricular junction and in the posterior mitral leaflet, 2D-ECHO	The duration of hemodialysis therapy was significantly longer in patients with MAC compared to those without MAC (13.4 ± 8.6 years vs. 7.7 ± 8.4 years; p<0.01), and an association was observed between the duration of HD and MAC (OR = 1.09, 95% CI 1.02–1.16; p<0.01) The number of coronary risk factors (SAH, dyslipidemia, smoking, and DM) was significantly higher in the MAC progression group compared to the non-progression group (1.91 ± 0.83 vs. 1.18 ± 0.99; p=0.03), and an association was noted between them (OR = 2.67, 95% CI 1.24–5.76; p=0.01)	MAC: 28 (29%), with transverse diameter of mitral annuls = 12.4 ± 7.4 mm
Xiong et al, 2022, China^21^	CVC by 2D-ECHO; 282 also underwent computed tomography. Calcified plaque if CT value greater than 130	HD duration ≥ 36 months (OR = 2.25; 95% CI 1.26–4.02, p=0.006), DM (OR = 1.81; 95% CI 1.04–4.12, p=0.037), low serum albumin levels (considering albumin ≥ 40 g/L vs < 40 g/L, OR = 0.54; 95% CI 0.29–0.99, p=0.047), serum calcium ≥ 2.11 mmol/L (OR = 2.04; 95% CI 1.01–4.12, p=0.046) and pulse pressure > 72 mmHg (OR = 3,22, 95%CI 1.85-5.59, p<0.001) were independently associated with CVC	CVC: 93; MVC: 37; AVC: 68; calcification of both valves: 12

AS: aortic stenosis; CTA: computed tomography angiography; MAC: mitral annulus calcification; CAPD: continuous ambulatory peritoneal dialysis; CT: computed tomography; CVC: cardiac valve calcification; DM: diabetes mellitus; APD: automated peritoneal dialysis; AP: alkaline phosphatase; GCCS: Global Cardiac Calcium Score; PPG: peak pressure gradient; SAH: systemic arterial hypertension; HD: hemodialysis; PP: pulse pressure; CRP: C reactive protein; T-PAFT: thoracic periaortic fat; the level of significance was set at p < 0.05, except for the study by Engole et al.,^15^which did not specify the p-value; aOR: adjusted odd ratio.

The most common associated factor was advanced age, found in 10 publications.^10 – 19^According to Alamir et al.,^10^there was a positive association starting at 55 years old (OR = 4.01, 95% CI 2.55-6.32). An association above 60 years was observed in the studies by Engole et al.^15^(ORa = 4.48; 95% CI 1.67-30.10, p=0.003), Guo et al.^16^(r = 0.25; p=0.003), Guerraty et al.^12^(moderate/severe VCA, respectively, 62.26 ± 7.9 and 66.53 ± 7, p<0.001), and Sayarlioglu et al.^19^(60.3 ± 13.5 years, p<0.001); as well as Rong et al.^13^above 70 years (70.42 ± 11.83 years, p<0.001; OR = 1.091, 95% CI 1.048-1.136, p<0.05). Some studies did not establish a reference value.^11 , 14 , 16 – 18^

Low glomerular filtration rate (GFR), in the absence of RRT,^10 , 12 , 20 – 23^was another associated factor, as was a GFR < 50mL/min/1.73m^2^in the in the study by Alamir et al.,^10^(ORa = 2.30; 95% CI 1.40-3.79), or a GFR less than 45mL/min/1.73m^2^in the study by Asselbergs et al.^20^(OR = 1.15; 95% CI 1.03-1.12, p=0.015) and Guerraty et al.^12^(p<0.0001). Fox et al.^22^reported an association with a GFR = 78 ± 25mL/min/1.73m^2^(p<0.001), a level considered reduced based on normal GFR values (90mL/min/1.73m^2^),^4^but above the cutoff value for determining CKD. Cystatin C was associated with mitral annulus calcification (MAC) in the study by Asselbergs et al.^20^(OR = 1.12; 95% CI 1.03-1.23; p=0.013).

In patients undergoing HD or PD, the chronicity of these procedures was also associated with Cardiac Valve Calcification (CVC).^3 , 16 , 19 , 21 , 24^Sayarlioglu et al.^19^observed that patients with CVC had undergone HD for a longer time (19.6 ± 40.6 months vs. 7.1 ± 5.8 months in non-RRT patients, p=0.01). Xiong et al.^21^found a significant association between CVC and HD lasting more than 36 months (OR = 2.25; 95% CI 1.26-4.02, p=0.006). Usuku et al.^24^demonstrated a similar result for Mitral Valve Calcification (MVC), comparing groups that started RRT earlier (13.4 ± 8.6 years vs. 7.7 ± 8.4 years, OR = 1.09; 95% CI 1.02–1.16; p<0.01). Tian et al.^3^specifically analyzed PD, finding a positive association for durations exceeding 20 months (OR = 1.039; 95% CI 1.004-1.075; p=0.03).

Reduced serum albumin levels were associated with CVC in several studies,^3 , 13 , 16 , 18 , 19 , 21 , 25^particularly among patients undergoing RRT. Rong et al.^13^linked CVC to pre-albumin levels of 238.44 ± 91.48 g/L, p=0.05. According to Plytzanopoulou et al.,^18^this decrease is a positive predictor of CVC, with an AUC of 0.73, 95% CI 0.57–0.89, p=0.012. Chen et al.^25^identified higher serum albumin levels in patients with CVC at more advanced stages of CKD (36.61 ± 4.37 g/L, p<0.05).

The only inflammatory marker associated with CVC was C-reactive protein (CRP).^12 – 14 , 18^Guerraty et al.^12^demonstrated a direct correlation between CRP levels and the severity of aortic valve calcification (AVC), with levels of 4.85 ± 7.29 mg/L for moderate AVC and 5.48 ± 8.77 mg/L for severe AVC, p=0.01. Rong et al.^13^observed that patients with any CVC had higher CRP levels (mean value of 5.5, ranging from 0.5–16.35 mg/L, p=0.04). Among studies involving patients on RRT, Ávila-Díaz et al.^14^found an association with RR = 1.09, 95% CI 1.04–1.19, p=0.04, while Plytzanopoulou et al.^18^reported an AUC = 0.692, 95% CI 0.58–0.89, p=0.03.

Alterations in mineral and hormonal factors appeared frequently in the studies. Serum levels of phosphate,^3 , 10 , 11 , 15 , 23^calcium,^17 , 21 , 25 – 27^CaxP product,^11 , 15^PTH,^14 , 16 , 25 , 26^FGF-23^23 , 25 , 26^and the Klotho cofactor^23 , 25^were directly and positively associated with CVC.

## Discussion

Several predictors of valvular calcification and valvular diseases were identified in patients with CKD. Advanced age was the most cited, with cutoff values above 55 years. The results were similar in studies that separately calculated the likelihood of MVC and AVC, allowing the conclusion that aging similarly affects both valves, consistent with other studies.^28 , 29^Even in patients without CKD, age is relevant to the development of valvular calcification of degenerative etiology, with a prevalence of 10% in the elderly population,^30^and is also a traditional risk factor for other cardiovascular conditions.^9^

The progressive and irreversible reduction of GFR, persisting for at least three months and at levels below 60 mL/min/1.73m^2^,^2^was one of the criteria for diagnosing CKD.^4^In this systematic review, studies showed an association between GFR and CVC starting at 45-50 mL/min/1.73m^2^.^10 , 12 , 20^The duration of RRT and hypoalbuminemia were also identified as risk factors. Most studies investigated the possibility of an association between the chronicity of HD and CVC,^16 , 19 , 21 , 24^with the duration ranging between 19.6 months and 13.4 years. London et al.^31^stated that dialysis mechanisms promote a repetitive cycle of valvular stress due to blood flow speed and the degree of turbulence. Regarding the comparison between HD and PD, Wang et al.^29^stated that cardiovascular conditions are similar between patients in both modalities. Conversely, Mary Laxton,^32^in a review article, pointed out that PD might slow the calcification process by preserving some residual kidney function, outperforming HD. It cannot be confirmed that the higher frequency of findings related to HD in this systematic review reflects a greater risk of this modality, as approximately 90% of the population under any RRT undergoes it.^33^

This systematic review observed an association between low serum albumin levels (<3.5 g/dL) and CVC in patients undergoing RRT. Santos et al.^34^concluded that, beyond malnutrition, factors such as reduced synthesis rate due to metabolic acidosis, inflammation, insufficient protein intake, hemodilution, and the type of membrane used in RRT (especially when sterilized with sodium hypochlorite) contribute to the reduction of serum albumin in patients on HD.^34^Endothelial dysfunction and atherosclerosis secondary to the action of acute-phase proteins and inflammatory cytokines could also explain CVC.^9^It is worth noting that Wang et al.^35^observed that patients with CVC undergoing PD were significantly more malnourished and had lower serum albumin levels compared to those without CVC, and the prevalence of CVC was higher as albumin levels decreased. Extrapolating beyond CVC, hypoalbuminemia is a well-known independent predictor of cardiovascular events in general, as well as mortality and chronic systemic inflammation in CKD.^9^

Current scientific evidence points to a positive relationship between elevated inflammatory markers and CKD, as observed by Shankar et al.,^36^who evaluated IL-6, TNF-α, CRP, and white blood cell count, with the strongest association for TNF-α. In this systematic review, three markers were associated with CVC – CRP, IL-6, and TNF-α, with only the first also reported in patients undergoing RRT. It is well known that inflammation is common in the later stages of CKD and that the calcification process originates mainly in areas under chronic inflammation, as observed in the genesis of atherosclerotic plaques.

Secondary hyperparathyroidism due to CKD appears to be related to the development of CVC. Hyperphosphatemia, resulting from the inability to excrete phosphate, is associated with calcification of both the mitral and aortic valves.^37^Hypercalcemia and elevated levels of FGF-23 have also been studied in CKD patients. In this systematic review, these indicators were associated with CVC. In CKD, the loss of nephrons impairs the kidney's ability to excrete phosphates, leading to increased formation of calcium-phosphate complexes. The resulting reduction in ionized calcium levels stimulates calcium-sensitive receptors in the parathyroid glands to secrete PTH to increase renal excretion of these components. However, PTH raises serum calcium by stimulating reabsorption from bones and kidneys. Concurrently, the conversion of vitamin D to calcitriol further increases intestinal absorption of both ions. These disorders ultimately result in extracellular calcium deposition.

This systematic review has some limitations: some of the included studies had small sample sizes and short follow-up periods. Only studies with observational methodologies were included, most of which were cross-sectional, limiting causal inferences. The studies also presented variations in analyzed populations, comparison groups, inclusion and exclusion criteria, as well as in the methods for measuring outcomes, scoring systems, and statistical techniques, which made adequate comparison of results challenging.

This heterogeneity constitutes a limitation as it compromises the comparability and external validity of the findings. However, this does not invalidate the findings of the systematic review. The heterogeneity of the results obtained can be explored through appropriate statistical analyses, such as random-effects models in meta-analyses, presenting an opportunity for new investigations and providing directions for future research. Different methodologies offer complementary perspectives, enriching the understanding of the topic, with dozens of risk factors identified by the included studies. Regular monitoring and vigilance of these parameters in clinical practice can enable the early identification of CKD patients at risk of valvular calcification and valvular diseases, allowing better opportunities for prevention and treatment, ultimately leading to increased survival and improved quality of life.

## Conclusion

This study identified several risk factors for the development of valvular calcification in patients with CKD. The main ones were age, advanced stages of the disease, duration of RRT, hypoalbuminemia, cytokines and other inflammatory agents, as well as products involved in the metabolism of secondary hyperparathyroidism.

Data Availability

The underlying content of the research text is contained within the manuscript.
